# Evaluating the impact of media and feed combinations on CHO cell culture performance and monoclonal antibody (trastuzumab) production

**DOI:** 10.1007/s10616-024-00690-7

**Published:** 2025-01-09

**Authors:** Aron Gyorgypal, Antash Chaturvedi, Viki Chopda, Haoran Zhang, Shishir P. S. Chundawat

**Affiliations:** Department of Chemical and Biochemical Engineering, Rutgers, The State University of New Jersey, Piscataway, NJ 08854 USA

**Keywords:** Mammalian cell culture, Chinese hamster ovary (CHO) cells, Media-feed optimization, Monoclonal antibodies (mAb), N-linked glycosylation

## Abstract

The choice of media and feeds significantly influences the performance of Chinese Hamster Ovary (CHO) mammalian cell cultures in producing desired biologics like monoclonal antibodies (mAb). Sub-optimal nutrient feed/media composition can severely impact cell proliferation and the quality of the final mAb product. For instance, proper protein glycosylation, crucial for mAb stability, safety, and efficacy, heavily relies on cell culture conditions. Currently, starter CHO culture media and daily supplemental feeds used in industrial manufacturing consist of proprietary composition of nutrients critical for mAb production. Standardized optimal media/feed combinations necessary for different cell lines are often lacking, necessitating individualized optimization for each cell line and mAb product. Here, we focused on a CHO-K1 cell line engineered to produce a Trastuzumab biosimilar and evaluated the effects of fourteen commercially relevant basal media and seven feeds on cell culture parameters such as viable cell density, viability, nutrient consumption, metabolite production, mAb titer, and mAb N-glycosylation. Our findings demonstrate clearly that the compositions of the basal medium and feed play a pivotal role in enhancing cell growth and mAb production. This work offers valuable insights into strategies for optimizing feed/media composition for glycosylated monoclonal antibody production using CHO cells.

**Supplementary Information:**

The online version contains supplementary material available at 10.1007/s10616-024-00690-7.

## Introduction

Monoclonal antibodies (mAbs) contribute to a large fraction of therapeutics on the market today (Ecker et al. 2015). Though many cell lines can be engineered to produce mAbs, Chinese Hamster Ovary (CHO) cells have been adopted as the primary workhorse in protein-based biologics manufacturing. Efficient and robust cell cultivation is required to ensure the desired and consistent quality of products. To this end, bioprocess outputs are defined by culture parameters, such as viable cell count (VCC), cell viability, mAb production rate, and key metabolite concentrations (e.g., glucose, lactate, ammonia) (Alt et al. 2016; Yuk et al. 2011), which are impacted by cultivation conditions, including the basal/initial media composition, feed composition, feeding strategy, and the cell cultivation mode (i.e., batch, fed-batch, or perfusion) (Walther et al. 2019; Pollock et al. 2013). In particular, developing rational strategies for using feed/media in suitable combinations is essential for improving fed-batch cell cultures’ process efficiency (Shukla et al. 2017; Rasoul et al. 2019).

A typical fed-batch culture process starts with a starter culture with a specific viable cell count in a basal medium that is generally suitable for supporting cell growth and maintaining cell viability at the beginning of the cultivation process. Subsequently, cells are passaged in the same basal medium or a combination of the basal medium and a feed to adapt the cells for bioreactor cultivation. Feed is added at certain time intervals to maximize the volumetric productivity and product titer. Basal media and feeds composition often need to be optimized to facilitate cell growth, protein productivity, gene expression, product quality (Pan et al. 2017).

On the other hand, culture feed/media formulation and optimization are complex and time-consuming. Cell cultures require a balanced supply of micronutrients such as vitamins, amino acids, and trace metals to support proper cell growth and high-quality glycoprotein production. As a result, chemically defined media for CHO cells are widely used for biomanufacturing. For example, some chemically defined media were developed to achieve high cell density, while others were designed to extend cell viability or enhance cell-specific productivity. Hence, the choice of basal medium and feed composition differ widely depending on the requirements of specific biomanufacturing processes, such as the type of CHO cell line used, characteristics of the generated subclones, the target bioproducts, and several other factors (Rodrigues et al. 2012; Reinhart et al. 2015). Thus, there is no single basal medium or feed suitable for all CHO cell lines, and for each CHO cell culture, the adopted basal medium feed and corresponding feeding strategies need to be specifically screened, designed, and optimized to produce the mAb product with targeted Critical Quality Attributes (CQAs).

**Fig. 1:**
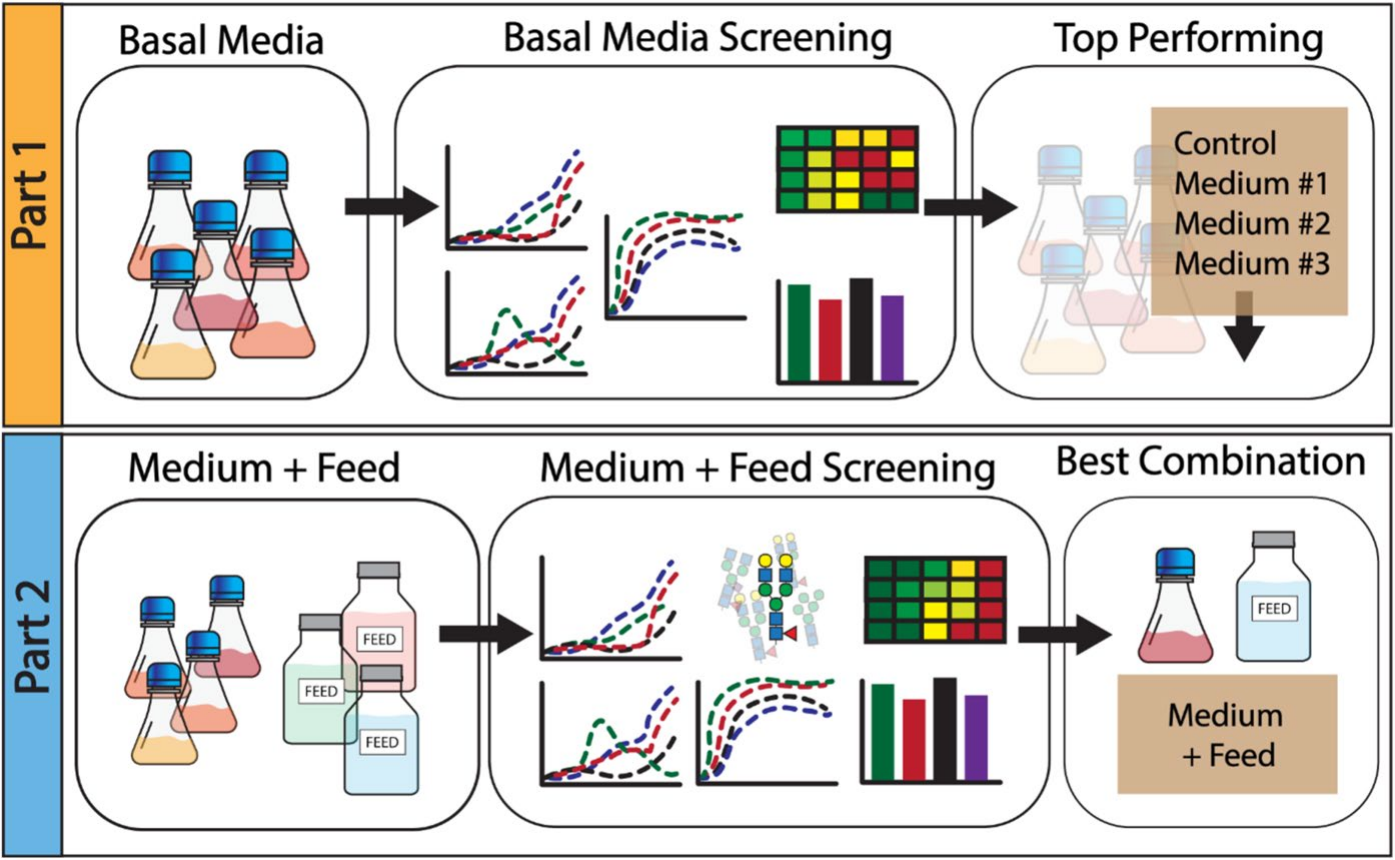
Overview of the experimental design for screening (**Part 1**) basal media by batch cultivation and (**Part 2**) different basal media-feed combinations for fed-batch cultivation. Key production parameters tested here, include VCC, viability, metabolite accumulation, productivity, and mAb N-glycosylation for comparative evaluation

This study investigates how different basal media and medium-feed combinations affect cell culture growth, metabolism, and mAb production of a CHO cell line engineered to produce Trastuzumab biosimilar. Figure 1 shows a schematic design of the overall outline of this study. In part 1, we first systematically investigated the effect of 14 basal media on cell culture growth performance cultivated in batch mode. Cell viability, and concentrations of key metabolites, such as glucose, lactate, ammonia, and mAb titer, were monitored for benchmarking performance of the adopted media. In part 2, we selected four basal media (the top three basal media from part 1 and CD-CHO™ as a control medium) and seven different feeds to make different combinations and evaluated their impact on bioproduction performance. We observed significant metabolic differences and bioproduction variations between the selected medium-feed combinations. In addition, a detailed amino acid analysis of the spent media revealed critical amino acid compositional changes during course of cell culture for production of the mAb product. The findings of this work highlight the importance of tailoring the medium-feed combination for improvements in mAb titer bioproduction, including CQAs like N-glycosylation.

## Materials and methods

### Cell line and culture medium

The Chinese Hamster Ovary-glutamine synthetase (CHO-GS) cell line was provided by GenScript (Piscataway, NJ). This cell line can adapt and grow in glutamine-free media since GS cell lines derive glutamine from the enzymatic conversion of glutamate and ammonia by the enzyme glutamine synthetase (GS). When the GS inhibitor methionine sulfoximine (MSX) is added to the medium, endogenous GS activity in CHO cells is inhibited to allow the survival of only GS transfectants. Hence, glutamine was excluded from the cell culture media to maintain this selection marker. The cell line was prepared in CD-CHO medium (Gibco), a chemically defined medium with 0.5% anti-clumping agent and 25 µM MSX (hereafter the combination called as CD-CHO basal medium).

Fourteen chemically defined basal media were used in this study: BalanCD CHO Growth A, IS CHO-CD G12.1, IS CHO-CD G17.1, IS CHO-CD G17.4, IS CHO-CD G17.6, IS CHO-CD G17.7 (from Irvine scientific, Santa Ana, CA); CD-CHO, CD-OptiCHO; CD-FortiCHO, and Dynamis (from Gibco, Thermo Fisher Scientific, Waltham, MA); and Ex-Cell Adv. CHO fed-batch, Ex-Cell CD CHO fusion, Ex-Cell CHO-5, and Ex-Cell CD CHO (from Sigma-Aldrich, St. Louis, MO). In addition, different feed media were used in this study: BalanCD CHO Feed 4, IS CHO-CD F12.7 (from Irvine scientific, Santa Ana, CA); Dynamis, Efficient A plus, B plus, and C plus (from Thermo Fischer Scientific, Waltham, MA); Ex-Cell Adv CHO feed-1 (from Sigma-Aldrich Aldrich, St. Louis, MO).

### Pre-culture and cell passaging

A vial of cells from a working cell bank was first thawed in CD-CHO basal medium (with 0.5% anti-clumping agent and 25 µM MSX) in a 125 mL unbaffled shake flask (VWR, Radnor, PA, USA) with 40 mL working volume. The cells were then grown at 37 °C, 130 RPM, and 5% CO_2_ in an orbital shaker for 4 days. Next, cells were passaged in a 250 mL shake flask with 100 mL working volume in CD-CHO basal medium. Once sufficient cell count was reached, the culture was transferred to a 50:50 mixture of CD-CHO and a selected basal medium. Note that 12 basal media selected for screening. Cultures were given two passages in this mixture medium for adaptation before transferring to a final 100% basal media under study. Before the basal media screening fed-batch experiments, cells were passaged twice in the 100% basal medium. For all experiments under evaluation, the final media consisted of a 0.5% anti-clumping agent and 25 µM Methionine sulfoximine (MSX).

### Batch and fed-batch culture cultivation

The culture was started with a viable cell count of 0.5 × 10^6^ cells per mL and propagated at 37 °C, 130 RPM, and 5% CO_2_ concentration in the incubator shaker for the batch culture experiments. Glucose was kept in the range of 3–5 g/L by adding 50% glucose solution daily as needed. The batch cultures were harvested when viability reached < 80%. For fed-batch experiments, 6% feed (v/v) was added on days 4, 6, 8, 10, and 12 and then harvested on day 14 or when viability < 80%, whichever is earlier.

### Cell density and viability measurements

Culture samples were taken daily from cell culture shake flasks. Cell density and viability were measured by the trypan blue exclusion method using a BioProfile FLEX-2 analyzer (Nova Biomedical, Waltham, MA, USA).

### Spent media analysis

Glucose, lactate, ammonia, glutamate, glutamine concentrations, pH, and osmolality were measured daily by a BioProfile FLEX-2 analyzer (Nova Biomedical, Waltham, MA, USA).

### mAb titer analysis

For mAb quantification, the culture samples were centrifuged and filtered through a 0.22 µm PVDF syringe filter (Celltreat. Pepperell, MA). The filtered samples were analyzed using an Agilent 1260 HPLC system with a Bio-Monolith Protein A analytical column (Agilent Technologies, Santa Clara, CA) following manufacturer’s instructions.

### Amino acid quantification

Amino acid sample preparation and analysis were conducted using an AccQ-Tag ultra-derivatization Kit (Waters Corp, Milford, MA). Briefly, 80 μL of AccQ Tag borate buffer was added to 10 μL of the culture samples in a total recovery HPLC vial, followed by the addition of 20 μL of AccQ-Tag reagent. The samples were then capped, vortexed, and incubated at room temperature for 1 min before transferring to a 55 °C heat block for 10 min. The prepared samples were then analyzed by HPLC following the manufacturer’s instructions.

### Glycan fraction analysis

The culture samples were purified by protein-A resin to separate targeted mAb from impurities. N-glycan profiles were determined using a deglycosylation and InstantPC (IPC) labeling kit GX96-IPC (Agilent Technologies, Santa Clara, CA), and glycan fractions were analyzed using an Agilent 1260 HPLC with an AdvanceBio glycan-mapping column (Agilent Technologies, Santa Clara, CA). Sample preparation and HPLC analysis were done following the manufacturer’s instructions for the IPC kit and HPLC column.

## Results and discussion

### Basal media screening

**Fig. 2:**
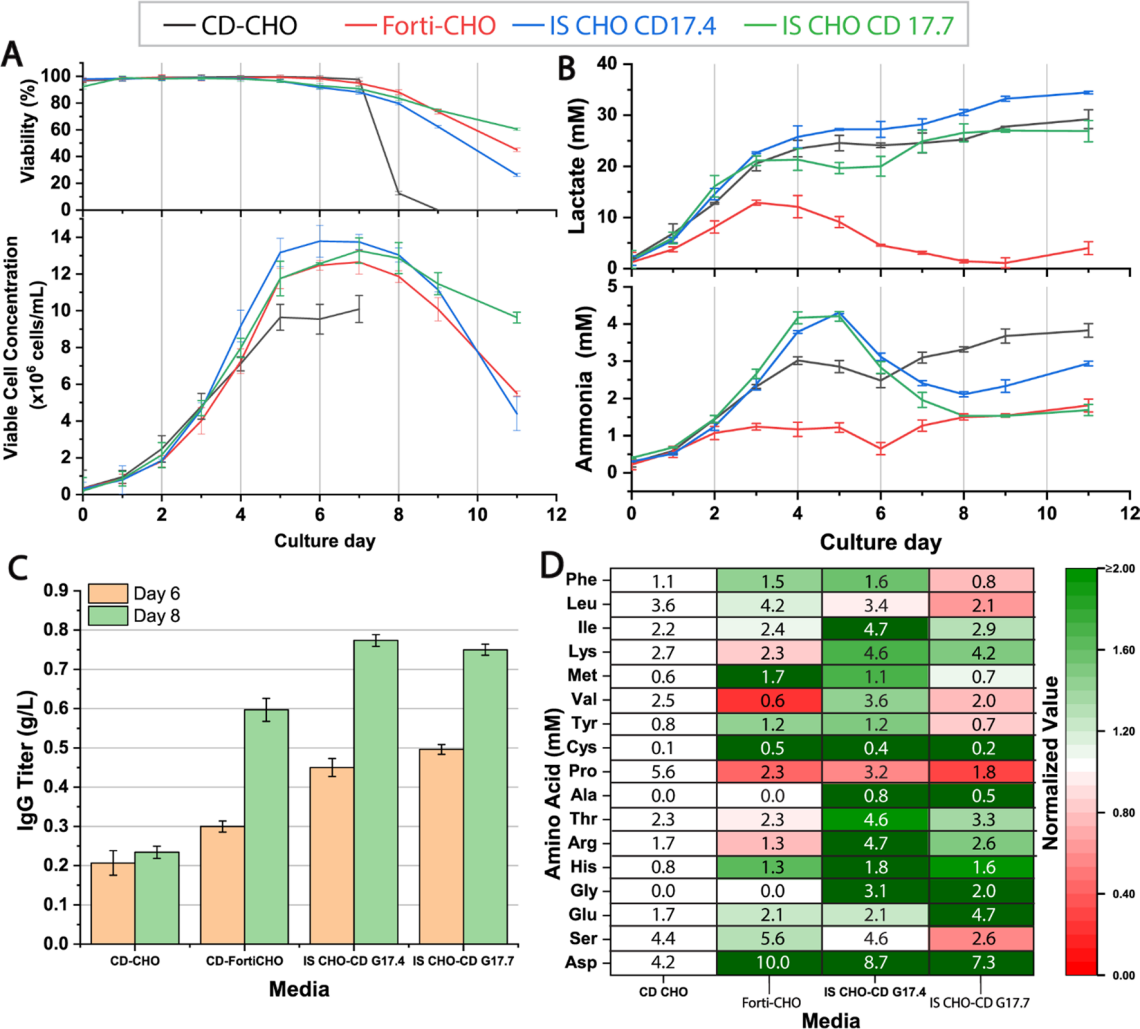
Comparison of basal media for impact on batch cultures’ (**A**) viability and VCC, (**B**) metabolites lactate and ammonia accumulation, and (**C**) mAb product titer on day 6 and 8. (**D**) Heat map shows concentrations of amino acids quantified in the basal media

Increasing product titer is one of the primary goals in biotherapeutics production. A higher titer is generally achieved by increasing cell count or cell-specific productivity. The selection of the basal media for the cell culture predominantly affects viable cell count, cell longevity, and specific productivity. We screened 14 basal media, with various compositions, including the control medium CD-CHO. Different performance parameters of bioproduction impacted by these media were plotted as outlined in Supplementary Fig. S1. We observed that the basal media IS CHO-CD G17.4, IS CHO-CD-17.7, and CD-FortiCHO yielded higher viable cell counts. Figure 2A shows the viable cell count profiles of the shake flask batch culture for the top three performing basal media versus the control medium CD-CHO. It was found that the use of IS CHO-CD G17.4, IS CHO-CD-17.7, and CD-FortiCHO media led to higher peak cell counts of around 13–14 million cells/mL, compared to only 10 million cells/mL in the control medium CD-CHO. Moreover, VCC in basal media Ex-Cell CD CHO fusion, Ex-Cell CD-CHO and Ex-Cell CHO 5 was below 5 million cells/mL (see Supplementary Fig. S1). In particular, VCC was only 1 million cells/mL in Ex-Cell CD-CHO and Ex-Cell CHO 5 media, indicating that the cells did not adapt well in these media. The average VCC was 8–10 million cells/mL for other basal media tested in our study.

The viability of shake flask batch culture cultivated in the top-performing media is shown in Fig. 2A. The viability in all other media is shown in Supplementary Fig. S1. For the basal media BalanCD CHO Growth A, CD-OptiCHO, and IS CHO-CD G17.1, the viability of the cell culture dropped below 80% on day 8. In comparison, we observed a rapid drop in cell viability in CD-CHO medium on day 7. Overall, we note that CD-FortiCHO, IS CHO-CD G17.4, and IS CHO-CD G17.7 are better at supporting cell growth to achieve a high viable cell count within the investigated cultivation time.

Glucose was the primary energy source for all media used in this basal media screening study. Glucose concentration was maintained between 3 and 5 g/L in the shake flask study, and the glucose concentration profile for all conditions is shown in Supplementary Fig. S1. The evaluated basal media significantly impacted CHO cell metabolism and resulted in varied accumulation of certain by-products such as lactate and ammonia which are known to affect cell growth above a certain threshold (Xing et al. 2008). In addition, changes in the by-product metabolites are also known to affect the pH of the internal Golgi body, which can largely influence the mAb product quality.

The lactate and ammonia concentration profiles for the top-performing media are shown in Fig. 2B and those profiles for other media are shown in Supplementary Fig. S1. It was found that lactate levels increased until day 5 when peak VCC was reached. Subsequently, the cells started to consume lactate when grown in all basal media except CD-CHO, IS CHO-CD G17.4, and IS CHO-CD G17.7. Using these three media led to higher lactate concentrations of 26.6, 34.4, and 28.9 mM, respectively, at the end of the cultivation, accompanied by ammonia accumulation of 3.8, 4.3, and 4.3 mM, respectively. On the other hand, the lactate and ammonia levels in the CD-FortiCHO medium were < 12.2 mM and < 2 mM, respectively, lower than most other media.

The pH and osmolality profiles of the shake flask basal media screening cultures are shown in Supplementary Figure S1. Note that pH was not controlled in these screening studies but was recorded for monitoring purposes. Osmolality level is a good indicator of nutrient consumption and for basal media Ex-Cell CD CHO fusion, Ex-Cell CD-CHO, and Ex-Cell CHO 5 that were tested, the osmolality was relatively high throughout the cultivation (around 300–340 mOsmol/kg). This indicates that the cells could not efficiently consume the fed nutrients, resulting in low VCC. On the other hand, a rise in osmolality was observed for the CD-CHO medium from 289 mOsmol/kg on day 6 to 308 mOsmol/kg on day 8, and there was a significant pH drop from day 6 (due to lactate accumulation), which is consistent with the decline in cell viability as shown in Supplementary Fig. S1. For the top-performing media, including CD-FortiCHO, IS CHO-CD G17.4, and IS CHO-CD G17.7, the osmolality steadily decreased from day 0 to day 8, which matched well with the higher VCC profiles.

The selection of basal media significantly impacted the mAb production. As cell viability dropped dramatically after day 8, the mAb concentrations on day 6 and day 8 were compared. As shown in Fig. 2C, the mAb concentration was higher in media with higher VCC. The mAb titer in IS CHO-CD G17.4, IS CHO-CD G17.7, and CD-FortiCHO basal media reached 0.77 g/L, 0.75 g/L, and 0.60 g/L, respectively. In comparison, the cell culture using CD-CHO produced only 0.25 g/L mAb. Rodrigues et al. compared the growth and mAb production in CHO-K1 cell line using five different media and also found that the mAb production by the CD-CHO media was often lower than other tested media (Rodrigues et al. 2012).

To further understand the cause for variable performance of basal media studied, we conducted a detailed amino acid analysis. Figure 2D shows a heatmap illustrating the amino acid distribution in all media tested. The average amino acid concentrations for each medium were normalized by that of the CD-CHO medium and presented in different colors to facilitate comparison. The effect of media amino acid composition on VCC and mAb production has been extensively studied before. Pereira et al. investigated the roles and contribution of various medium components on the CHO cell metabolism and found that serine was the most readily consumed amino acid by the CHO-GS cell line (Pereira et al. 2018). Selvarasu et al. performed an in-silico metabolic analysis of CHO cells and mouse hybridoma cells and found significant differences in the culture’s amino acid compositions, in both the growth and non-growth phases (Selvarasu et al. 2012). In our study, we note that three of our top-performing media had distinct amino acid compositions, especially for aspartate, serine, cysteine, histidine, and proline, whereas these amino acids showed sub-optimal concentrations in the control basal media likely causing lower VCC and mAb production. The results hereby suggest that these amino acids are beneficial for improving mAb production by the cell line used here.

### Feed-media combination screening and impact on cell growth performance metrics

Based on the basal media screening results, the top 3 basal media (based on improved performance in terms of cell growth, protein concentration, and other metabolite levels), which included IS CHO-CD G17.4, IS CHO-CD G17.7, CD-FortiCHO, were selected to further improve mAb production using fed-batch mode cultivation. CD-CHO was used as a control medium again for comparison. An initial viable cell count of 0.5 × 10^6^ cells/mL was used. The cell culture was cultivated under the same conditions described for the batch culture above, except that select feed media (6% v/v) were added on days 4, 6, 8, 10, and 12.

**Fig. 3:**
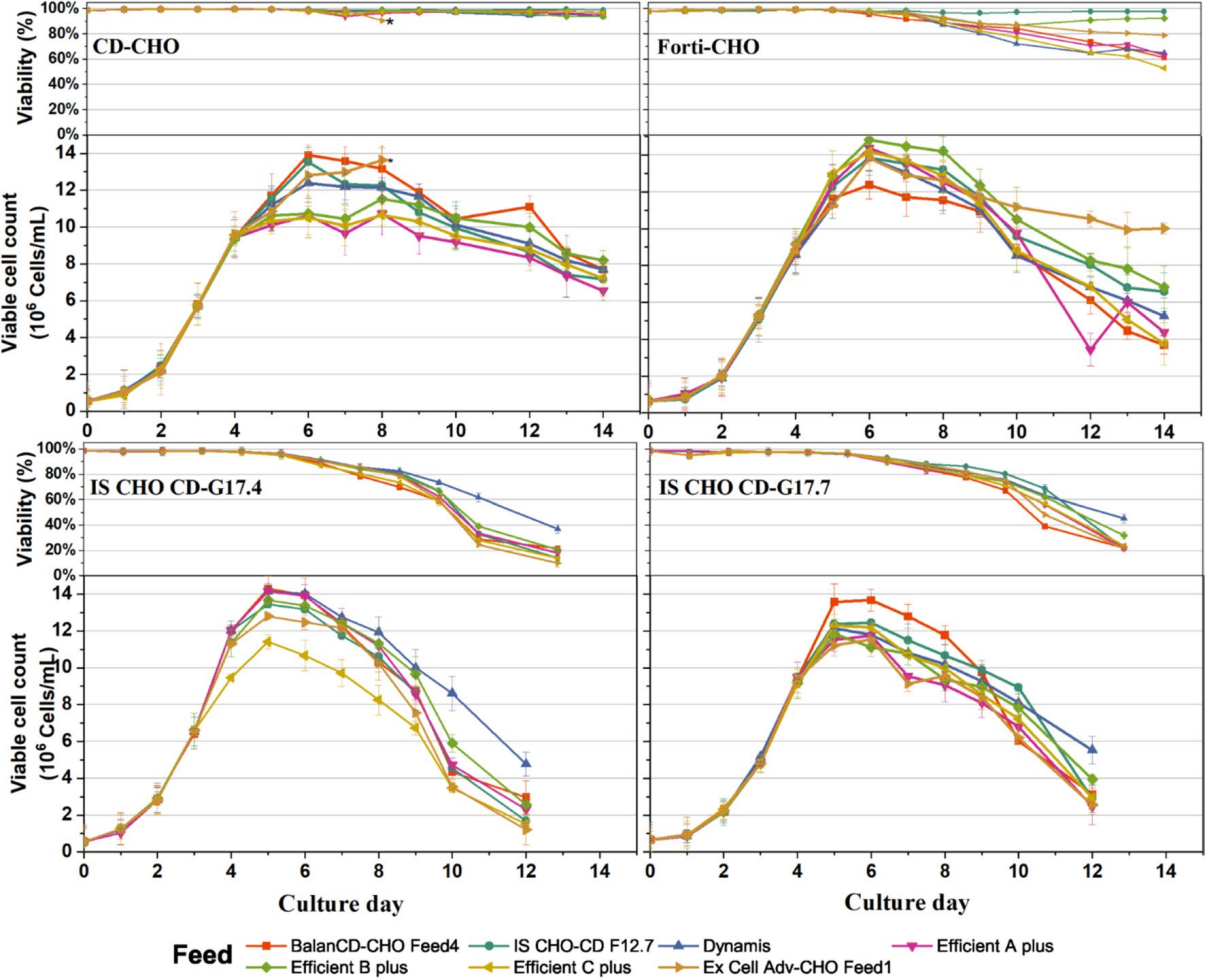
VCC and viability of cell cultures grown with different media-feed combinations

The impact of various media-feed combinations on viable cell counts and viability is shown in Fig. 3. IS CHO-CD G17.4 and IS CHO-CDG17.7, two top-performing media from the batch culture study in terms of VCC and titer, showed a marginal increase in the VCC with all the tested media-feed combinations. In contrast, CD-FortiCHO performed significantly better, as the highest VCC peaked at 14.5 × 10^6^ cells/mL in CD-FortiCHO + Efficient B plus and around 14.0 × 10^6^ cells/mL in CD-FortiCHO + Efficient (A plus and C plus). The control medium (CD-CHO) achieved peak VCC of 13.0 × 10^6^ cells/mL in CD-CHO + BalanCD CHO feed 4 and around 12.0 × 10^6^ cells/mL in CD-CHO + (IS CHO-CD F12.7/Dynamis/ Ex-Cell Adv-CHO feed 1). However, in CD CHO + Efficient (A plus / B plus/ C plus), only a marginal increase in VCC was observed compared to the CD-CHO batch culture.

For IS CHO-G17.4 and IS CHO-G17.7, the cell viability of the fed-batch culture with all seven different feeds dropped down to below 80% after 8 days. This is consistent with the findings from our batch culture study as well. Feed supplementation marginally improved the longevity of the culture grown in the CD-FortiCHO basal medium, for which the viability of all combinations was lower than 80% on day 11 except for two feeds. CD- FortiCHO, in combination with Efficient B plus and IS CHO-CD F12.7, maintained higher viabilities than the other two top-performing feeds. s, CD-CHO basal medium with all tested feeds maintained a viability of above 90% till day 14.

**Fig. 4:**
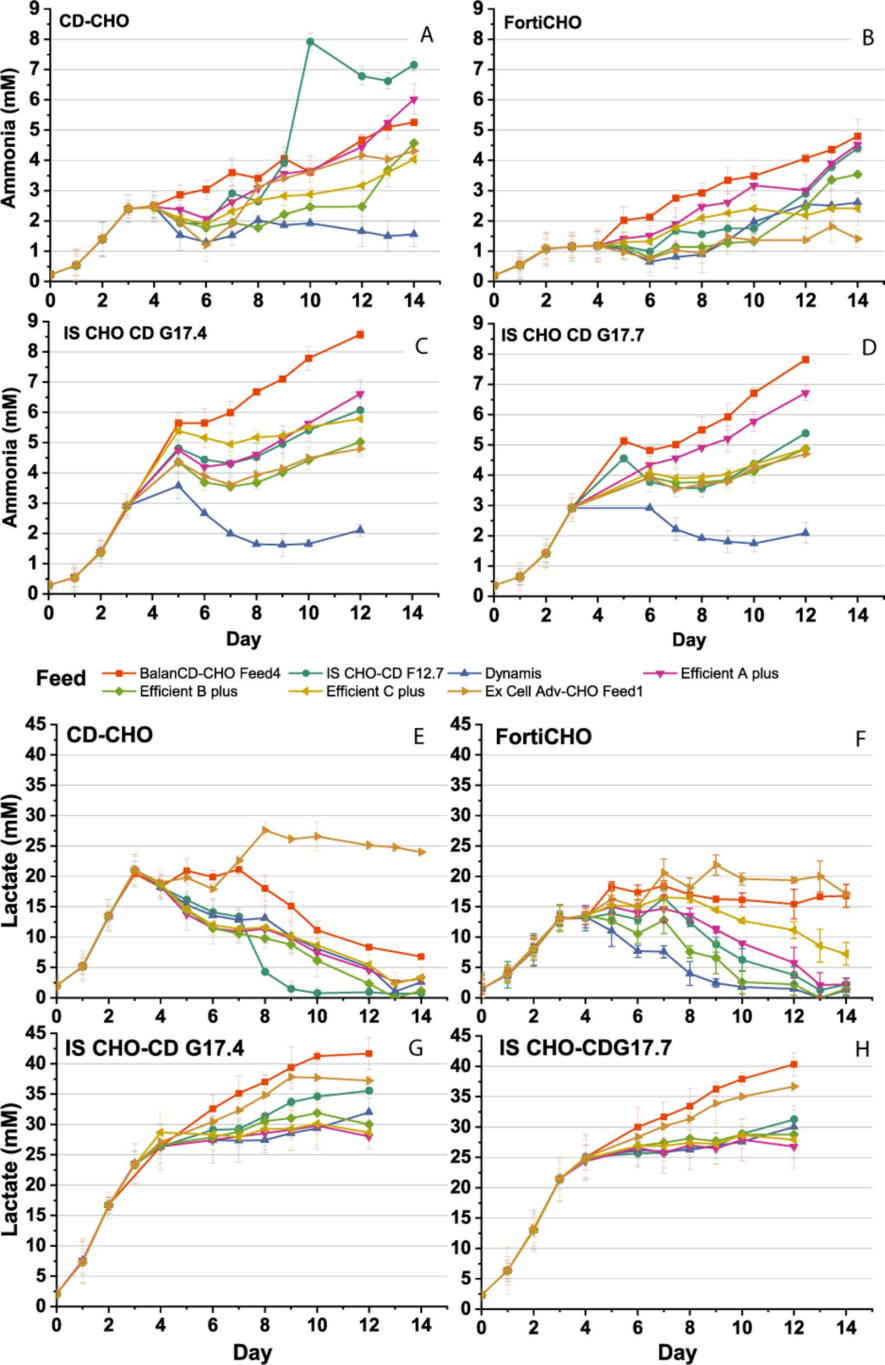
Accumulation of ammonia and lactate in cell cultures grown on different basal media-feed combinations

Figure 4A–H shows the lactate and ammonia concentration changes with culture duration for all basal media-feed combinations. In cultures using CD-CHO and CD-FortiCHO as the basal media, the by-product accumulation declined over time as shown in Fig. 4E, F, indicating the transition from lactate production to lactate consumption (Mulukutla et al. 2012). The BalanCD CHO Feed4 in both CD-CHO and CD-FortiCHO basal media showed a delayed transition from lactate production to consumption, which led to an increase in the overall lactate level in the culture. Using Ex-Cell Adv-CHO as the feed resulted in higher lactate accumulation in both CD-CHO and CD-Forti-CHO basal media. By the end of the cultivation, the lactate concentration was stabilized at 24 mM in CD-CHO and 16.6 mM in CD-FortiCHO, respectively. In the fed-batch cultures with IS CHO-CD G17.4 and IS CHO-CD-17.7 as the basal media, a continuous lactate accumulation (Fig. 4G, H) was observed with all seven feeds.

The ammonia concentration profiles showed varied trends for different basal media-feed combinations. The addition of BalanCD CHO Feed 4, IS CHO-CD F12.7, and Efficient A plus caused a higher accumulation of ammonia as shown in Fig. 4A–D. In contrast, the use of Dynamis feed led to lower levels of ammonia accumulation. All the other combinations followed a similar trend and remained below 10 mM. Notably, BalanCD CHO Feed 4 resulted in an elevated ammonia accumulation in all tested basal media, probably due to the high glutamate content in this feed (Hong et al. 2010).

The feeding strategy of fed-batch cultures inherently influences pH and osmolality. pH and osmolality changes are shown in the Supplementary Figs. S2, S3. It was observed that when CD-CHO was used as the basal medium, all the feed supplements except Efficient A plus and Ex-Cell Adv-CHO feed1 maintained a pH of around 7.1 ± 0.2. For Efficient A plus, pH dropped below 6.8 after day 6 and was maintained at this level until the end of the culture. For the cell culture grown in CD-FortiCHO, three feed supplements, namely Dynamis, Efficient B plus, and IS CHO-CD-F12.7, maintained a stable pH of around 7 (Supplementary Fig. S2). For the fed-batch cultures using basal media IS CHO CD-G17.4 and IS CHO CD-G17.7, pH gradually shifted to 6.7–6.3, consistent with the finding of lower pH in batch cultures after day 6 (Supplementary Fig. S3).

A considerable difference was observed in osmolality for different feeds used with CD-CHO and CD-FortiCHO cultures. In particular, BalanCD-CHO Feed4 resulted in osmolality greater than 400 mOsm/kg on day 13. The CD-FortiCHO culture fed with Efficient B plus showed a declining trend of osmolality, which went under 230 mOsm/kg on day 6 and declined further to 200 mOsm/kg by the end of the cultivation. This could be due to the higher nutrient consumption with the adopted feeding strategy. However, for IS CHO-CD G17.4 and IS CHO-CD G17.7 cultures, osmolality remained between 250 and 340 mOsm/kg except for BalanCD-CHO Feed4, where it reached 400 mOsm/kg on day 11 (Supplementary Figs. S2, S3).

**Fig. 5:**
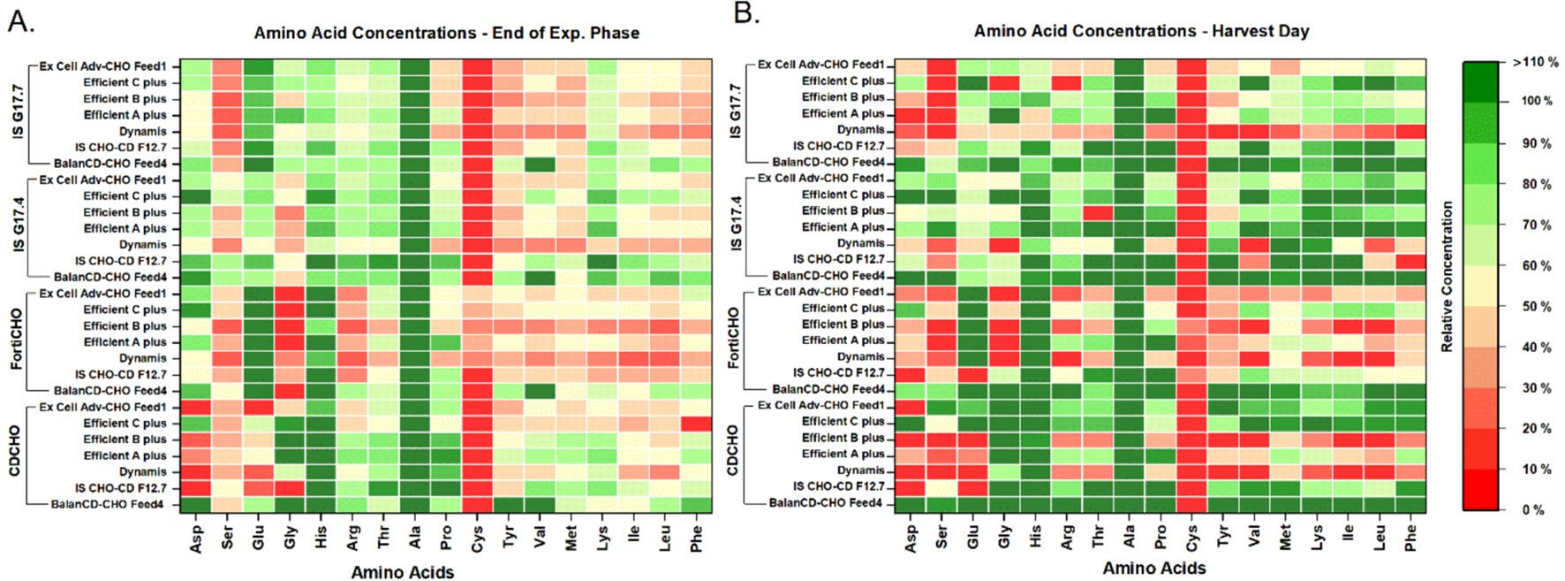
Fed-batch cultures’ amino acid profiles at (**A**) the end of exponential phase and (**B**) the end of cultivation

**Fig. 6:**
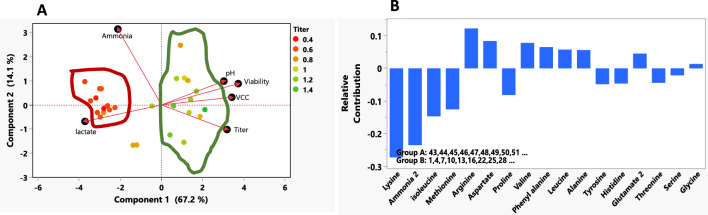
Score cum loading plot using Principal Component Analysis (PCA) for later days of cell culture and relative contribution plot of amino acids for the two identified groups in the score cum loading plot

To investigate how the basal medium selection resulted in different growth and bioproduction performance, amino acid analysis was conducted on spent media, and the measurements were normalized by the initial amino acid concentrations at the beginning of cultivation. Based on our analysis, cysteine and serine were consumed the most during the fed-batch run (Fig. 5). However, it should be noted cysteine is known to degrade quickly, which may interfere with the quantification of this amino acid (Calvet and Ryder 2014). Figure 6A shows the differences in the fed-batch process performance evaluated using principal component analysis (PCA) of various process performance parameters such as VCC, viability, lactate, ammonia, pH and titer. We observed that the basal media IS CHO-CD G17.4 and IS CHO-CD G17.7 with all feed conditions showed higher lactate and ammonia accumulation which subsequently resulted in lower VCC, viability and lower titer. A similar phenomenon was noted when CD-CHO and Forti-CHO media were supplemented with Excell Adv-CHO Feed1. To understand this further, we have evaluated the amino acid levels during the process, and found that these differences can be attributed to the differences in the compositions of basal medium and feed at amino acid levels as shown in Fig. 6B which depicts the relative differences in amino acid composition among two marked groups in the Fig. 6A.

### Feed-media combination screening and impact on mAb titer and quality metrics

**Fig. 7:**
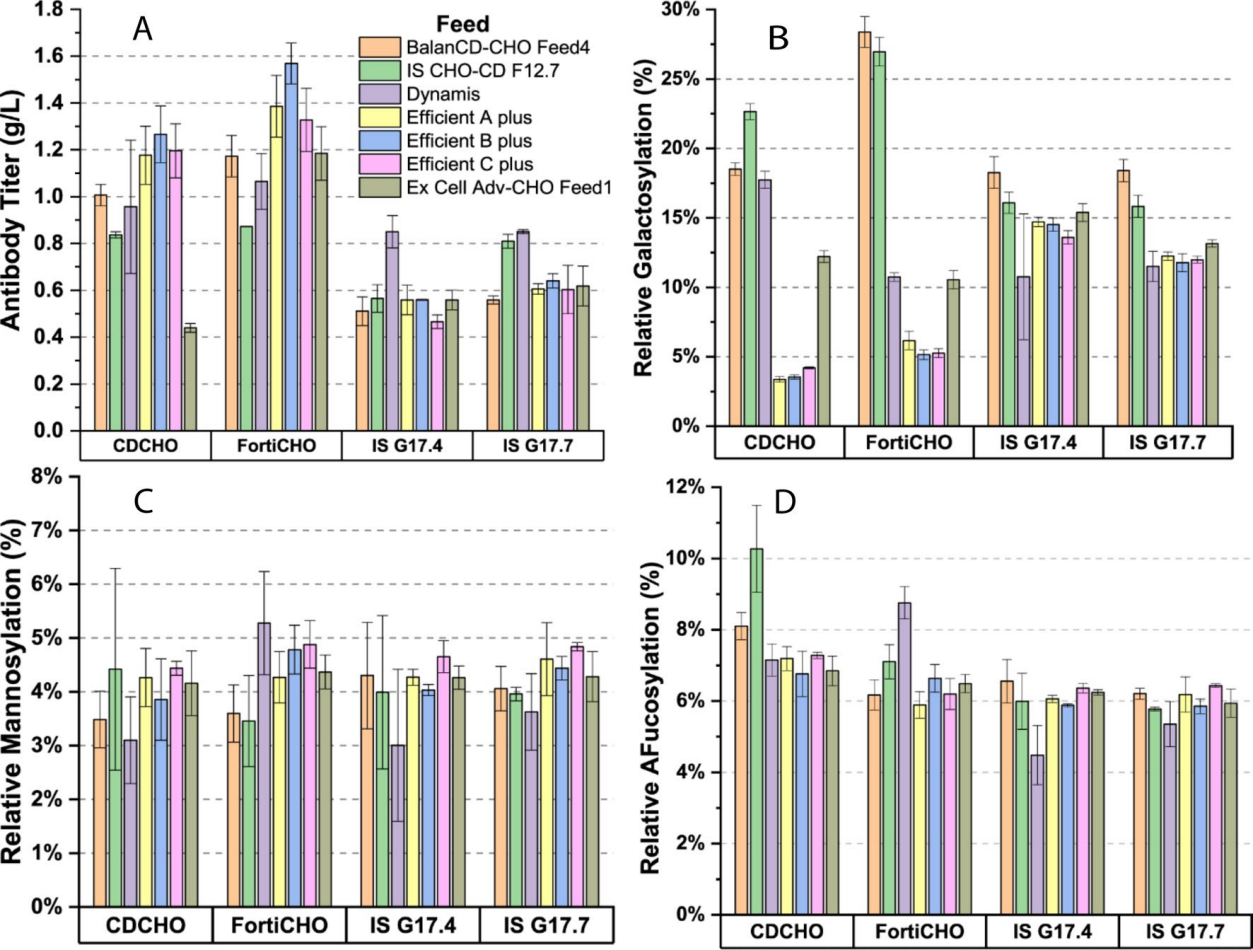
Analysis of fed-batch cultures produced mAb critical quality attributes, (**A**) protein titer, (**B**) galactosylation composition, (**C**) mannosylation composition, and (**D**) afucosylation composition

The combination of basal medium and feed significantly impacted the mAb product titer and quality attributes, such as the N-glycosylation profile. As shown in Fig. 7, CD-FortiCHO outperformed other basal media overall. CD-FortiCHO combined with Efficient B plus achieved the highest VCC of 14.8 × 10^6 cells/mL and a mAb titer of 1.63 g/L. In contrast, using different feeds like IS CHO-CD G17.4 and IS CHO-CD G17.7 did not significantly improve peak VCC, mAb titer, or viability compared to the batch cultures. This suggests that lactate and ammonia accumulation in these cases might influence productivity and peak viable cell count.

**Fig. 8:**
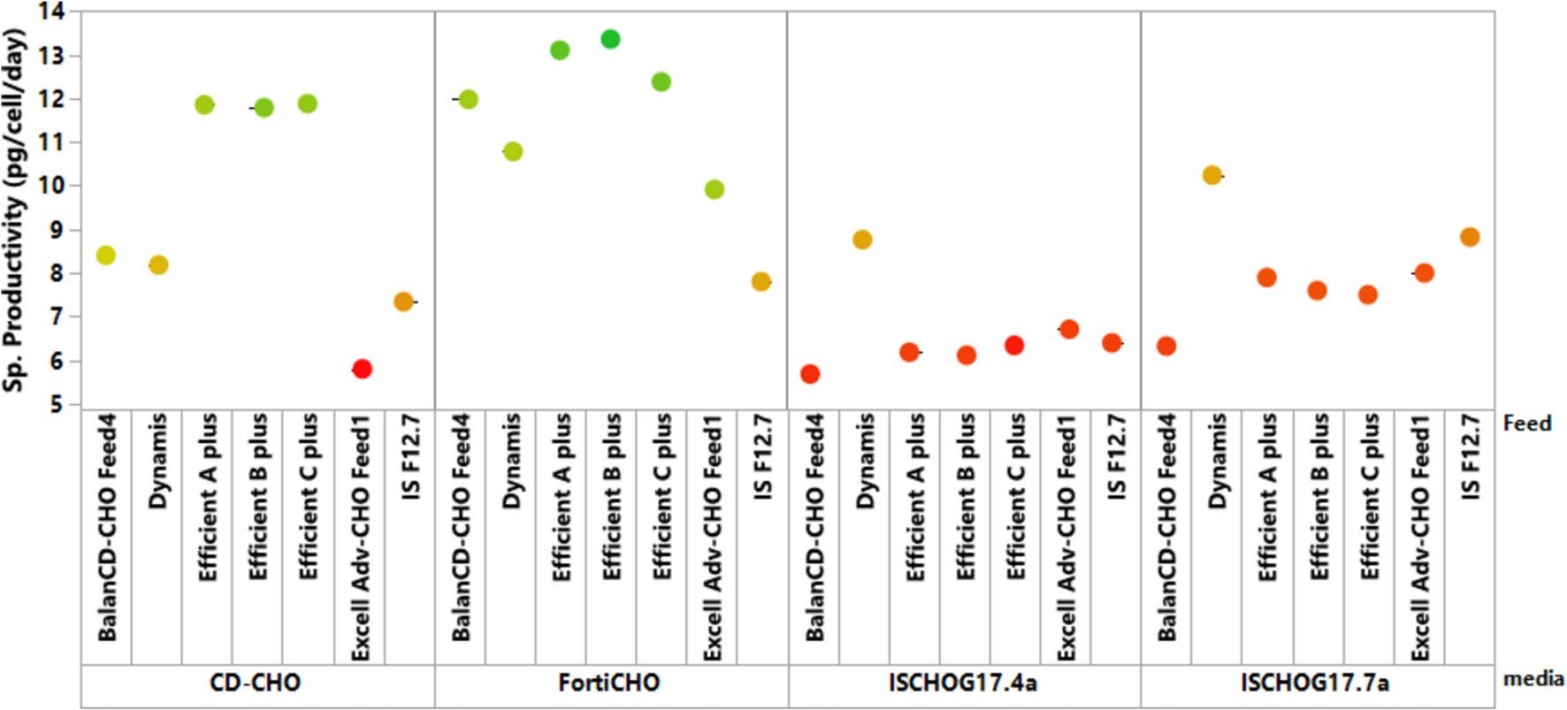
Comparison of specific productivity for evaluated media feed combination

In the fed-batch culture, CD-CHO with BalanCD-CHO Feed4 produced a higher VCC, but a higher product concentration was achieved with CD-CHO and Efficient B plus combination (1.35 g/L). This could be because Efficient B plus maintained a relatively hypoosmotic environment throughout the fed-batch process, which enhanced nutrient transport and improved culture longevity, as reported by Sue et al. (Sue Lee and Min 2001). Comparison of specific productivity has been depicted in the Fig. 8. It can be observed that media CD-CHO and/or FortiCHO in combination with feed of Efficient series showed higher specific productivity in the range of 12–14 pg/cell/day.

Given that N-glycosylation is a critical quality attribute or CQA determining the efficacy of the mAb product, we performed detailed glycan profiling to investigate the distribution of all major mAb glycoform products. Glycosylation for Trastuzumab using shake flask cultures is expected to be sub-optimal due to the lack of proper process controls at this scale. Nonetheless, mAb glycosylation using shake flask cultures can demonstrate variations among different basal media-feed combinations, which was previously also used to indicate the profound impact of selecting suitable media and feed combinations on final drug product quality (Lee et al. 2018).

The total galactosylation, afucosylation, and mannosylation levels were calculated for each fed-batch shake flask culture based on the glycoform compositional data. Supplemental Table S1 provides the detailed breakdown of glycoforms detected for each basal media and feed combination tested. The most abundant glycans were G0F (64.4%–88.4%), Man5 (2.4%–5.6%), G1F (3.5%–17.6%), and G0F-GN (0.6%–9.6%). A similar result was found in previous studies by Costa et. al (Costa et al. 2013) and Reinhart et al. (Reinhart et al. 2015). As shown in Fig. 7B, the three top performers for galactosylation were CD-FortiCHO with BalanCD-CHO Feed 4 or with IS CHO-CD F12.7, and CD-CHO with IS CHO-CD F12.7. The total galactosylation levels for these top performers were 27.7%, 26.9%, and 23.0%, respectively. In comparison, the control culture CD-CHO with Dynamis feed had a total galactosylation of only around 13%. A recent paper on glycosylation profiles of mAbs produced from CHO cells showed that the optimal value for galactosylation was often between 22.3% and 49.3% for the United States FDA approved Herceptin (Lee et al. 2018). Also, all four basal media supplemented with IS CHO-CD F12.7 as the feed showed an increase in galactosylation. This can be correlated with higher glutamate concentrations in these feeds, which has been shown to improve mAb galactosyaltion (Hong et al. 2010). Using Efficient A, B, and C plus as the feed supplements for the CD-CHO and Forti-CHO basal media generated a substantial decrease in total galactosylation and increased the truncated G0F-GN glycoform. The underlying mechanism for this result remains unclear. However, it is hypothesized that mono-glycosylated glycans can lead to protein misfolding, decreasing overall biologic efficacy (Parodi 2000). Our results showed that mAb galactosylation could be manipulated using different basal media and/or feed supplements, which is consistent with previous reports (Wells et al. 2020; Radhakrishnan et al. 2017). On the other hand, only minor variations were seen for mannosylation (Fig. 7C) and afucosylation (Fig. 7D), although CD-CHO with IS CHO-CD D12.7 resulted in 2.5% higher afucosylation than the control CD-CHO with Dynamis. It should be noted that it is possible to modulate these feeds’ impact on bioproduction by adding additional quality modifiers such as galactose, manganese, and/or uridine to improve the relative galactosylation abundance of the final drug product (Wells et al. 2020; St. Amand et al. 2014; Gramer et al. 2011). Moreover, further process optimization can be done by adding chemical modulators to control the CHO cell physiology and mAb production performance (Kildegaard et al. 2016; Madabhushi et al. 2021; Handlogten et al. 2018).

## Conclusion

In this study we evaluated various basal media-feed combinations by examining key performance indicators such as viable cell count (VCC), viability, monoclonal antibody (mAb) concentration, and mAb N-glycosylation. The media CD-FortiCHO, IS CHO CD G17.4, and IS CHO CD G17.7 demonstrated high performance in terms of VCC and titer during the batch culture cultivation. In the fed-batch cultivation, different feeds significantly influenced cell growth and mAb bioproduction behavior. Some feeds, including Efficient A plus, B plus, and C plus were beneficial for mAb productivity but resulted in sub-optimal galactosylation, a critical quality attribute for trastuzumab production. Therefore, these feeds may be suitable for mAb production requiring lower galactosylation. Additionally, other methods such as adding galactose, MnCl2, or adjusting physical parameters, e.g., pH and temperature, can be also used to modulate galactosylation. Our findings lay a foundation for further enhancing the bioproduction of trastuzumab and other glycosylated mAbs in both batch and fed-batch modes.

## Supplementary Information

Below is the link to the electronic supplementary material.

Supplementary file1 (PDF 760 KB)

## Data Availability

No datasets were generated or analysed during the current study.
