# Supplementary material for: Evaluating the impact of media and feed combinations on CHO cell culture performance and monoclonal antibody (trastuzumab) production
Source: Cytotechnology. 2025 Jan 9;77(1):40. doi: 10.1007/s10616-024-00690-7 (PMC11718031; doi:10.1007/s10616-024-00690-7)
Supplement: Supplementary file 1 — Supplementary file1 (PDF 760 KB) [file 10616_2024_690_MOESM1_ESM.pdf]

Supplementary Information (SI) Appendix

Evaluating the Impact of Media and Feed Combinations on CHO Cell Culture Performance and Monoclonal Antibody (Trastuzumab) Production

Aron Gyorgypal[1,#], Antash Chaturvedi[1,#], Viki Chopda[1,#], Haoran Zhang[1], Shishir P.S. Chundawat[1,*]

[1]Department of Chemical and Biochemical Engineering, Rutgers The State University of New Jersey, School of Engineering, Piscataway, New Jersey, United States 08854, USA

***Corresponding Author:** Shishir P.S. Chundawat (shishir.chundawat@rutgers.edu) ORCID: 0000-0003-3677-6735

#. All authors contributed equally

Supplementary Figure Legends:

Figure S1. Key cell culture performance metrics (i.e., Viable Cell Count, Viability, Ammonia concentration, Lactate concentration, Glucose Concentration, pH, Osmolality) for 14 Basal Media screened in batch mode.

Figure S2. Key cell culture performance metrics (i.e., pH, Glucose Concentration, Osmolarity) for CD CHO and Forti-CHO basal media with various feeds in fed-batch mode.

Figure S3. Key cell culture performance metrics (i.e., pH, Glucose Concentration, Osmolarity) for IS CD-CHO G17.4 and IS CD-CHO G17.7 basal media with various feeds in fed-batch mode.

Supplementary Table Legend:

Table S1. Average mAb glycoform distribution as a function of all media and feed combinations tested under fed batch cell culture conditions. Increase in galactosylation levels was a major difference between combinations tested as well as increase in truncated G0F-GN glycoforms when using efficient plus series (A, B, & C) feeds.

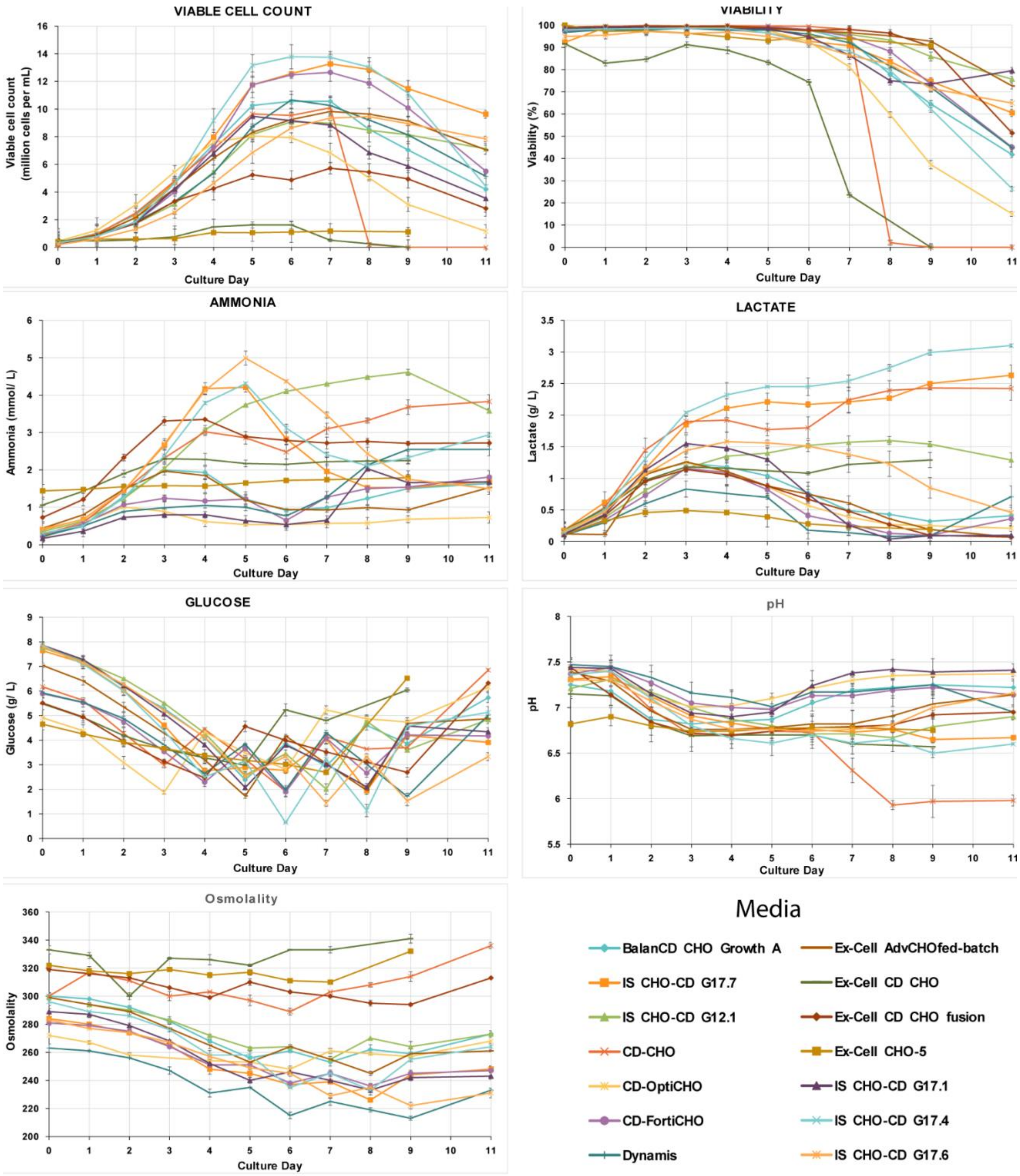

Supplementary Figure S1. Key cell culture performance metrics (i.e., Viable Cell Count, Viability, Ammonia Concentration, Lactate Concentration, Glucose Concentration, Culture pH, Osmolality) for 14 basal media screened in batch mode.

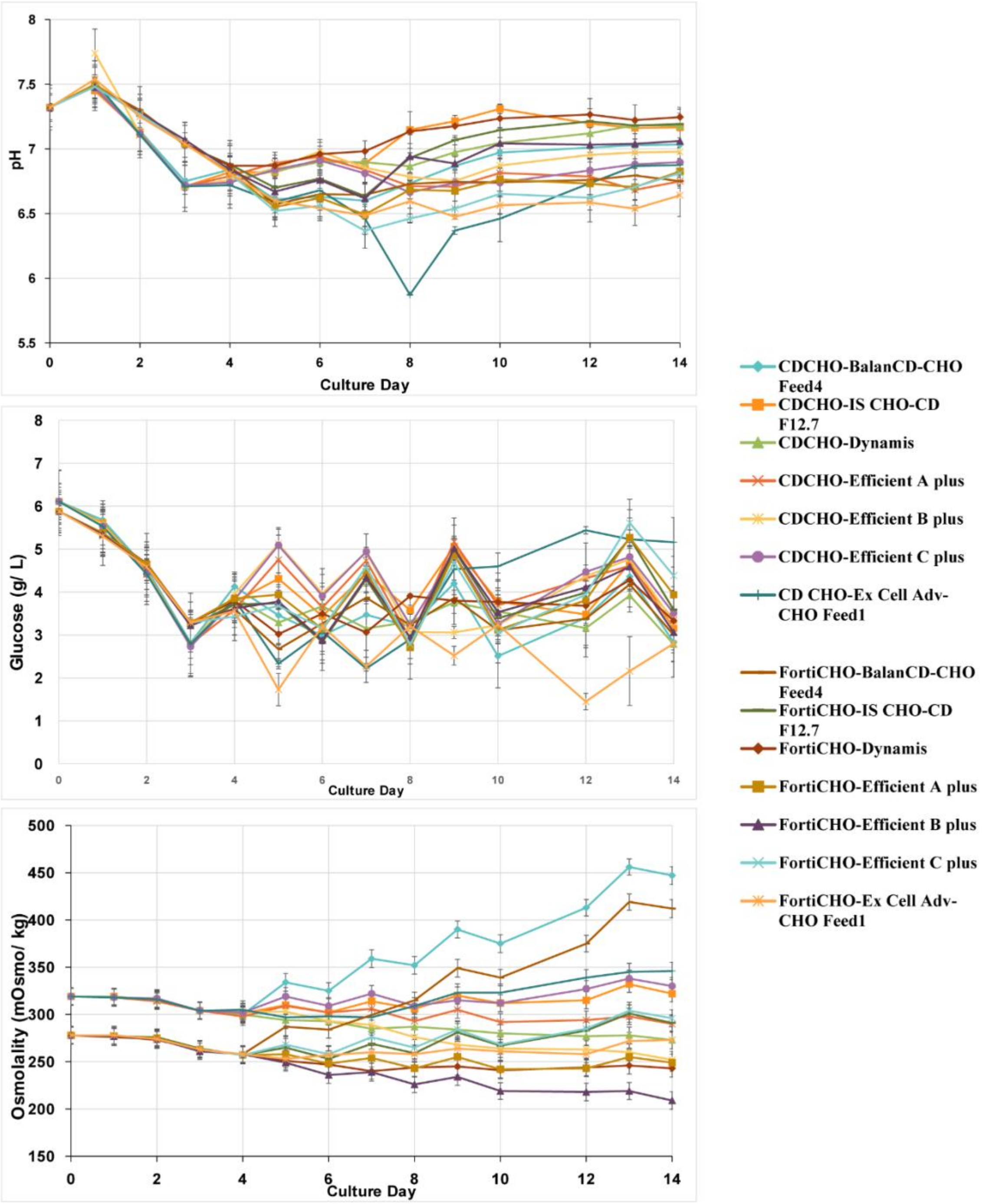

Supplementary Figure S2. Key cell culture performance metrics (i.e., pH, Glucose Concentration, Osmolarity) for CD CHO and Forti-CHO basal media with various feeds in fed-batch mode.

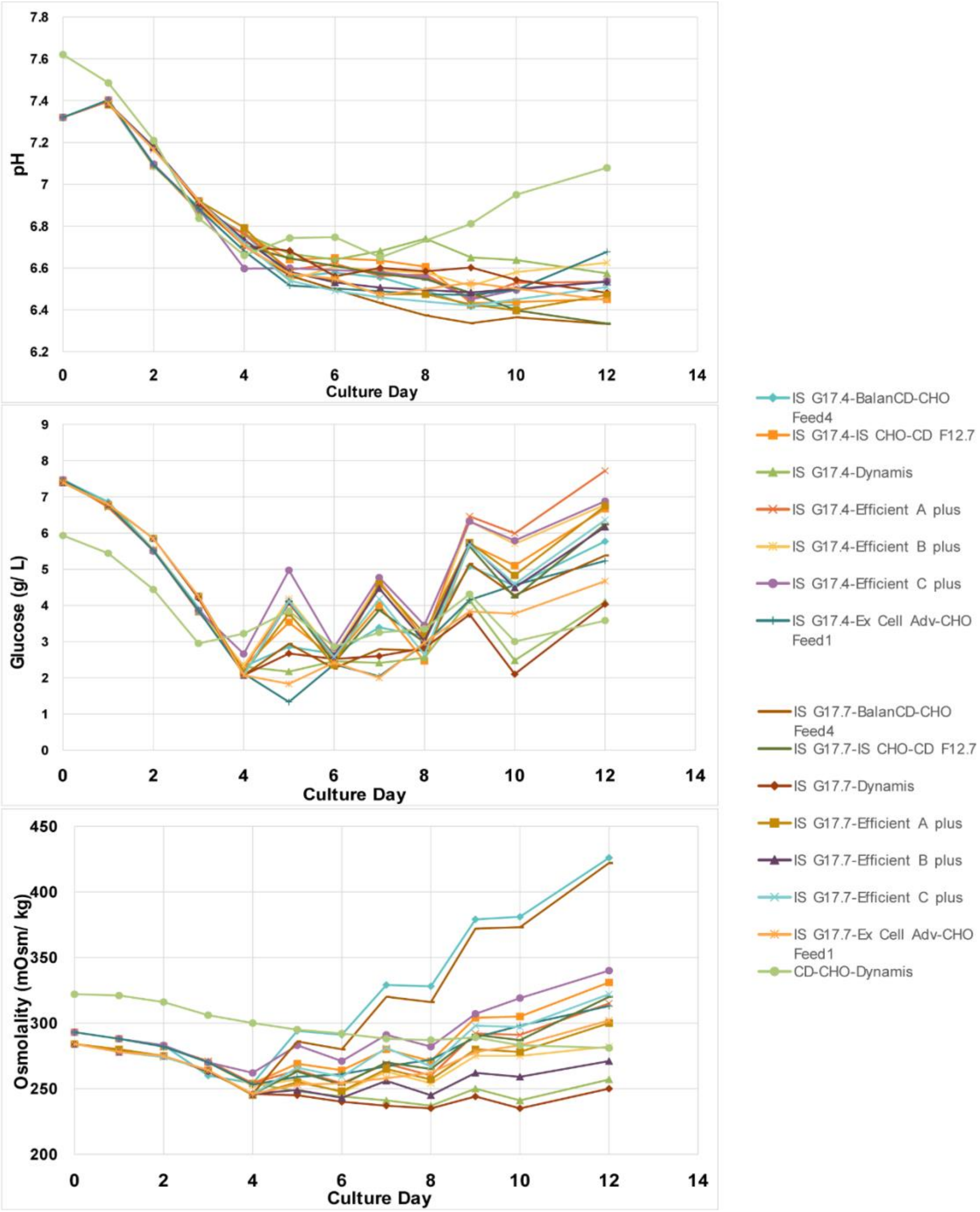

Supplementary Figure S3. Key cell culture performance metrics (i.e., pH, Glucose Concentration, Osmolarity) for IS CD-CHO G17.4 and IS CD-CHO G17.7 basal media with various feeds in fed-batch mode.

Media	Feed	mAb Glycoforms Relative Composition										
		G0F-GN	G0	G0F	M5	G1	G1F	G1F’	G2F	Total Galactosylation	Total Afucosylation	Total Mannosylation
CD-CHO	Dynamis	2.6%	4.0%	77.1%	3.5%	0.3%	8.5%	3.3%	0.8%	13.0%	7.8%	3.5%
	BalanCD-CHO Feed4	2.3%	4.2%	71.1%	3.8%	0.6%	11.8%	4.7%	1.5%	18.7%	8.5%	3.8%
	IS CHO-CD F12.7	1.9%	4.8%	65.0%	5.3%	1.5%	14.2%	5.3%	2.0%	23.0%	11.6%	5.3%
	Efficient A plus	5.6%	2.8%	83.7%	4.5%	0.0%	2.5%	1.0%	0.0%	3.5%	7.3%	4.5%
	Efficient B plus	6.5%	3.0%	82.6%	4.3%	0.0%	2.6%	0.9%	0.0%	3.6%	7.3%	4.3%
	Efficient C plus	6.8%	2.8%	81.7%	4.5%	0.0%	3.1%	1.1%	0.0%	4.2%	7.3%	4.5%
	Ex Cell Adv-CHO Feed1	4.2%	2.0%	77.4%	4.5%	0.5%	7.7%	2.9%	0.8%	11.9%	7.0%	4.5%
Forti-CHO	Dynamis	3.5%	3.3%	76.9%	5.6%	0.5%	6.6%	2.7%	0.8%	10.6%	9.4%	5.6%
	BalanCD-CHO Feed4	1.8%	2.2%	64.4%	3.9%	0.5%	17.6%	6.6%	3.1%	27.7%	6.5%	3.9%
	IS CHO-CD F12.7	1.7%	3.0%	64.6%	3.8%	0.9%	17.7%	5.7%	2.7%	26.9%	7.7%	3.8%
	Efficient A plus	4.9%	1.7%	84.5%	4.5%	0.0%	3.3%	1.1%	0.0%	4.4%	6.2%	4.5%
	Efficient B plus	9.6%	1.8%	78.7%	5.1%	0.0%	3.5%	1.4%	0.0%	4.9%	6.9%	5.1%
	Efficient C plus	5.1%	1.3%	83.3%	5.2%	0.0%	3.6%	1.4%	0.0%	5.0%	6.5%	5.2%
	Ex Cell Adv-CHO Feed1	2.1%	1.6%	81.6%	4.5%	0.4%	6.2%	2.6%	0.8%	10.1%	6.6%	4.5%
IS G17.4	Dynamis	0.6%	1.0%	88.4%	2.4%	0.1%	4.9%	1.9%	0.6%	7.6%	3.5%	2.4%
	BalanCD-CHO Feed4	1.0%	1.9%	74.5%	4.7%	0.7%	11.2%	4.5%	1.5%	17.9%	7.3%	4.7%
	IS CHO-CD F12.7	1.3%	2.5%	76.2%	4.5%	0.0%	10.2%	4.0%	1.3%	15.5%	7.0%	4.5%
	Efficient A plus	1.3%	1.5%	78.4%	4.3%	0.3%	9.3%	3.6%	1.3%	14.5%	6.1%	4.3%
	Efficient B plus	1.3%	1.5%	78.8%	4.1%	0.4%	8.9%	3.9%	1.1%	14.3%	5.9%	4.1%
	Efficient C plus	1.5%	1.8%	78.6%	4.7%	0.0%	8.7%	3.3%	1.2%	13.3%	6.6%	4.7%
	Ex Cell Adv-CHO Feed1	1.3%	1.5%	77.7%	4.3%	0.4%	10.1%	3.5%	1.2%	15.1%	6.2%	4.3%
IS G17.7	Dynamis	1.3%	1.7%	82.2%	4.1%	0.0%	7.2%	2.6%	1.0%	10.7%	5.7%	4.1%
	BalanCD-CHO Feed4	1.3%	1.9%	74.6%	4.2%	0.4%	11.6%	4.5%	1.5%	18.0%	6.5%	4.2%
	IS CHO-CD F12.7	1.1%	1.9%	77.8%	4.0%	0.0%	10.1%	4.0%	1.2%	15.3%	5.9%	4.0%
	Efficient A plus	1.6%	1.7%	79.6%	5.0%	0.0%	8.1%	3.0%	1.0%	12.1%	6.7%	5.0%
	Efficient B plus	1.3%	1.4%	81.2%	4.6%	0.0%	7.5%	2.7%	1.2%	11.4%	6.0%	4.6%
	Efficient C plus	1.6%	1.6%	80.1%	4.9%	0.0%	7.8%	2.9%	1.1%	11.8%	6.5%	4.9%
	Ex Cell Adv-CHO Feed1	1.4%	1.6%	79.4%	4.6%	0.0%	8.5%	3.2%	1.3%	13.0%	6.2%	4.6%

Supplementary Table S1. Average mAb glycoform distribution as a function of all media and feed combinations tested under fed batch cell culture conditions. Increase in galactosylation levels was a major difference between combinations tested as well as increase in truncated G0F-GN glycoforms when using efficient plus series (A, B, & C) feeds.
